# Sobriety and Satiety: Is NAD+ the Answer?

**DOI:** 10.3390/antiox9050425

**Published:** 2020-05-14

**Authors:** Nady Braidy, Maria D. Villalva, Sam van Eeden

**Affiliations:** 1 Centre for Healthy Brain Ageing, School of Psychiatry, University of New South Wales, Sydney, NSW 2052, Australia; m.villalva@unsw.edu.au; 2 Centre for Cutaneous Research, Blizard Institute, Barts and The London School of Medicine and Dentistry, Queen Mary University of London, London E1 4NS, UK; s.vaneden@qmul.ac.uk

**Keywords:** NAD+, cocaine, addiction, alcohol, cellular energetics

## Abstract

Nicotinamide adenine dinucleotide (NAD+) is an essential pyridine nucleotide that has garnered considerable interest in the last century due to its critical role in cellular processes associated with energy production, cellular protection against stress and longevity. Research in NAD+ has been reinvigorated by recent findings that components of NAD+ metabolism and NAD-dependent enzymes can influence major signalling processes associated with the neurobiology of addiction. These studies implicate raising intracellular NAD+ levels as a potential target for managing and treating addictive behaviour and reducing cravings and withdrawal symptoms in patients with food addiction and/or substance abuse. Since clinical studies showing the use of NAD+ for the treatment of addiction are limited, this review provides literature evidence that NAD+ can influence the neurobiology of addiction and may have benefits as an anti-addiction intervention.

## 1. Addiction: Today’s Most Common Modern Disease

Addiction represents the most common untreatable disorder in the 21st century. Addiction has a profound effect on individuals, their family and carers, and represents a major socio-economic burden on today’s healthcare system. Some epidemiological studies have suggested that addiction may be endemic in some populations [1]. The most common forms of addiction are alcoholism and smoking, both of which have been associated with increased risk of developing age-related disorders which manifest in the cardiovascular and central nervous system [2,3,4,5]. Tobacco smoking is the most preventable cause of morbidity and mortality worldwide. It has been estimated that of the 38 million people in the United States of America alone who attempt to quit smoking, more than 85% relapse within the first few weeks following withdrawal interventions. Abstinent smokers who successfully withdraw in the first few months are susceptible to relapse after six months or even years later [6]. Alcoholism is the third leading cause of death after cardiovascular disease and cancer and is associated with over 2.3 million deaths worldwide every year. Alcoholism is the ninth major contributor to global disease burden as measured by the disability-adjusted life years (DALYs) [2,3,4,5]. As with other forms of addiction, relapse occurs in response to exposure to environmental stimuli that are linked to the rewarding effects of the stimulant. Moreover, substance abuse (e.g., cocaine and heroin), internet addiction and excessive gambling are growing problems among the younger generation [7].

Food addiction has been associated with obesity, a global complex health problem that is dependent on multidisciplinary treatment and various health practitioners including experts in mental health, medicine and surgery [1]. Obesity has been associated with addictive behaviour that has a profound effect on morbidity and mortality leading to a reduction in the overall quality of life. The World Health Organization estimates that by the year 2030, over 57.8% of the world’s population will be obese. Obesity represents the second cause of death in the United States alone, affecting nearly 35 million people alone. Obesity has a negative effect on metabolic and endocrine processes that can lead to multiple organ disorders, malignant diseases such as cancers, mechanical impairments, surgical complications, and psychosocial deficits [8,9,10,11]. Obesity is also associated with genetic predisposition, impaired hormonal and metabolic processes, as well as confounding psychological and lifestyle factors [12,13,14,15]. This suggests that obesity is not a single disease, but rather a manifestation of several pathological, physiological and psychological processes leading to endocrine-metabolic dysfunction.

Substantial research into the pathobiology and mechanisms of addiction, and addiction therapy are yet to yield effective responses. The most successful addiction programs demonstrate a relapse-response rate of 56% in subjects [16,17,18,19,20]. Given the importance of improving the prevention and successful treatment of addiction, there is a growing need to investigate new factors that may be associated with addictive behaviour. Various experimental approaches suggest that replenishment or augmentation of cellular levels of the essential pyridine nucleotide, nicotinamide adenine dinucleotide (NAD+) can provide ameliorative benefits and significantly lower relapse in addiction.

## 2. Neurobiology of Addiction

A wealth of evidence has identified several similarities between individuals who demonstrate addictive food behaviour and those that are diagnosed as addicted to substances. DiLeone et al. showed that individuals that are addicted to food describe similar processes (e.g., rewarding properties, withdrawal symptoms and overeating) to those addicted to drug and alcohol addicts [21,22]. Food in today’s contemporary society is rich in calories, saturated fats, sugars, synthetic additives (e.g., colours) and preservatives which are of low nutritional value and widely accessible. Compulsive over-consumption of foods rich in salt and additives or saturated with sugars and lots of calories has the greatest potential for addiction [23]. These foods are termed ‘hyper palatable’, and can induce hedonistic behaviours and inhibit negative thoughts similar to substance abuse [24,25]. This can induce excessive preoccupation of food and periodic eating of large amounts of food within a very short time frame. These events may occur either once a day or once a week, leading to feelings of culpability, ignominy and depression, triggering further over-consumption of foods due to increased emotional stress [26,27]. Like substance abuse and sex addiction, ‘over-eating’ represents an in-effective mechanism to overcome negative thoughts or mediate ‘self-control’ [1]. There are also various similarities between the molecular basis of food and drug addiction which are detailed below.

### 2.1. Hyper-Activation of the Glutaminergic System

Overactivation of the glutaminergic system has been shown to play an important role in substance abuse and obese conditions. In particular, it has been previously shown that treatment with N-methyl-D-aspartate receptor (NMDAR) antagonists can attenuate drug cue associations, and reduce cue-induced drug seeking and relapse-like behaviour [28]. Moreover, intracerebroventricular and lateral hypothalamic injections of glutamate, or its excitatory amino acid agonists, kainic acid, AMPA, and N-methyl-D-aspartate (NMDA), enhance food appetite in murine models, while the mGluR5 receptor antagonist, (R,S)-2-chloro-5-hydroxyphenylglycine, suppresses appetite [29]. However, alternative therapies are warranted since NMDAR antagonism can induce psycho-mimetic adverse effects (e.g., hallucinations, paranoia, cognitive deficits) due to NMDAR antagonism [30]. 

### 2.2. Impaired Mitochondria Function

There is also substantial evidence indicating that brain energy homeostasis is perturbed in substance abuse and metabolic syndrome. This includes impairments in glucose metabolism, increased oxidative stress and reduced cellular respiration [31,32,33,34,35]. However, very few studies are available in the literature that investigate mitochondrial changes in selected brain regions associated with the central reward pathway for substance abuse and food addiction. Recent studies have reported evidence of mitochondrial dysfunction in impaired motivation states such as depression, anxiety and stress [36,37]. For example, BCL-2, which regulates mitochondrial Ca^2+^ homeostasis, has been associated with impairments in mood, and increased BCL-2 has been reported to stabilise mood [38]. In addition, mutations in several mitochondrial genes have been associated with increased anxiety in several animal models [39]. Transcriptomic profiling of the nucleus accumbens (NAc)—an important brain reward nucleus—identified enrichment in gene ontology of mitochondria-related transcripts following repeated cocaine exposure [40]. Exposure to substances also impaired the activity and function of mitochondrial complex I, oxidative phosphorylation and ATP production, and mitochondrial membrane potential [31]. 

Furthermore, studies using functional magnetic resonance imaging (fMRI) reported increased activity in regions of the brain associated with motivation and reward when a sample of 48 women were drinking a chocolate milkshake or exposed to photographs of milkshake cups getting filled. Higher activity was observed in women with greater addiction scores, and less activation was reported in regions of the brain associated with inhibition of certain behaviours [25,26]. This suggests that these women were less able to control their behaviours when exposed to milkshakes as stimuli. Another study showed that food addiction was correlated with increased activation in the anterior cingulate cortex, medial orbitofrontal cortex and amygdala in response to eating. Greater activation was reported dorsolaterally in the prefrontal cortex and caudate nucleus, and less activation in the lateral orbitofrontal cortex, similar to drug addicts [25,26].

### 2.3. Increased Neuroinflammation and Kynurenine Pathway Activation

Numerous studies have shown that neuroinflammation can induce depressive symptoms and increase the likelihood of developing addiction [41,42,43,44,45,46]. Activation of the kynurenine pathway (KP) of tryptophan (TRYP) degradation has been thought to play a major role in drug-related conditioned behaviours [47]. Several products of the KP are neuroreactive. For example, quinolinic acid (QUIN) is an NMDAR agonist and its accumulation can lead to excitotoxicity and impaired glutaminergic transmission [48,49,50]. Kynurenic acid (KYNA) is an NMDAR antagonist that exerts neuroprotective effects on the brain and can counteract the cytotoxic effects of QUIN [51]. We and others have hypothesised that the QUIN/KYNA ratio represents a balance between neurodegeneration and neuroprotection in the brain which may be associated with immune activation [49,52,53]. Inhibition of kynurenine-3-monooxygenase (KMO) by Ro61-8048 and its prodrug JM6 has been previously reported to induce a metabolic shift in the KP towards KYNA, thus reducing glutaminergic/NMDAR activity [47]. KMO inhibition also abolished relapse-like alcohol drinking and alcohol and cocaine-seeking behaviours [47]. However, given that nicotinamide adenine dinucleotide (NAD+) represents the final end product of the KP, it is unclear whether NAD+ levels can be effectively maintained when KMO is inhibited [54]. 

### 2.4. Alterations in the Mesolimbic-Fronto Cortical Dopamine Pathway

The mesolimbic-fronto cortical dopamine (DA) system (composed of the mesolimbic and mesocortical DA systems) is an important pathway in brain reward [55]. The neurotransmitter, DA, is a key component in the brain reward system. DA has been associated with both drug and food addiction. Alcohol consumption has been reported to directly stimulate DA release and increase DA levels [56]. In addition, the behavioural rewards of nicotine are associated with DA release in the mesolimbic pathway [57], and lesions in the mesolimbic DA pathway have been reported to lead to a reduction in self-administration of nicotine [55]. Cannabinoid receptors have also been identified in regions of the brain associated with reward. The active component of cannabis, A’-tetrahydrocannabinol (A9-THC) has been shown to increase DA levels [58]. 

The brain reward system also plays a crucial role in food addiction, promoting adaptive behaviours (e.g., consuming palatable nutrients by linking them to pleasurable thoughts). Release of gastrointestinal (GI) hormones in response to nutrients in the GI tract, limit food intake through activation of the reward system by influencing motivational behaviour and its reward value [59]. For instance, reduced GI production of the fat specific satiety factor oleoylethanolamide (OEA) can induce high-fat-diet induced obesity in mice [60]. Negative feedback mechanisms also exist between the brain and the gut for sugars, consumption of which is dependent on taste and post-ingestive factors which have a direct effect on the brain reward system or mediating the production of endocrine factors that influence sucrose satiety and reward [61].

### 2.5. Dysregulation of Endocrine Factors

The neuropeptide and hormone oxytocin (OXT) has been thought to play a role in reward, stress, social and cognitive processes which are affected by addiction [62]. OXT is a nonapeptide that is secreted by the brain from oxytocinergic neurons in the paraventricular and supraoptic nuclei of the hypothalamus [63]. The association between OXT and addiction stems from similarities between behaviours reported in human addiction and social behaviours [64]. Increased OXT levels following exogenous OXT via intranasal administration has been shown to boost trust, generosity and social recognition in humans without adverse effects [65]. However, these effects are context or situation dependent. There is also evidence of a common neurobiology between social interaction and addition as demonstrated in prairie voles and rodents [66]. Several preclinical studies have also shown that OXT can reverse the neuroadaptations occurring with chronic drug and alcohol use [67]. 

The liver is thought to be involved in the production of endocrine factors that influence addiction [68]. Fibroblast growth factor 21 (FGF21) is a liver-derived hormone that has various physiological and pharmacological effects. FGF21 has been reported to normalise blood glucose levels in diabetic animals, promote fatty acid oxidation, prevent β cell dysfunction and reduce weight gain in the high-fat-diet induced mice models [69,70,71,72,73,74,75]. FGF21 has also been reported to reduce sweet consumption in mice and humans whereas FGF21 knockout increases sugar consumption in murine models [76,77,78]. Additionally, overproduction of FGF21 signals to the brain to regulate food intake, energy expenditure and fertility, and prevent age-related increases in weight gain and insulin resistance [68,76,77,78]. FGF21 gene therapy has been demonstrated to be a potential therapy for obesity and type 2 diabetes [79]. Recently, FGF21 has also been reported to inhibit alcohol consumption in mice [80]. The levels of FGF21 are increased in human plasma following acute alcohol consumption and sustained binge drinking [80]. Taken together, these studies suggest that FGF21 is an endocrine inhibitor of alcohol and sugar consumption in humans.

### 2.6. Importance of Circadian Rhythms

The underlying pathobiology of addiction and DA release may be regulated diurnally by interplay between circadian rhythms and metabolism. Circadian disruption has been reported to influence behavioural responses to substance abuse [81,82,83,84,85]. For example, mutations in circadian genes in mice mediate differential locomotor sensitisation and conditioned preference to cocaine [86]. It is thought that these circadian rhythms are controlled transcriptional-translational feedback loops and selected protein coding genes governed by the molecular clock. In particular, the main circadian transcription factors CLOCK and BMAL1 have been reported to couple with various intracellular metabolic signalling pathways [87]. Circadian rhythms can regulate DA synthesis and release, which are dependent on the availability and activity of tyrosine hydroxylase (TH), whose transcriptional regulation is dependent on CLOCK/BMAL1 [88].

### 2.7. Role of Endogenous Opiates

Additionally, substance abuse and/or food have been shown to activate endogenous opiates, which represent a group of peptides that are produced in several organs and the brain and pituitary glands in particular [89]. The endogenous opioid system influences the mesolimbic DA and the cortisol stress response, which are both implicated in addiction reward [90]. The opioid antagonist naltrexone has been shown to inhibit the desire for sweet, salty and fatty foods, and alcohol and drugs [91]. 

## 3. Historical Background of NAD+

Pellagra is a debilitating disorder caused by the deficiency of niacin and/or its precursor tryptophan. Symptoms of pellagra were first documented in 18th century by the Spanish doctor Gasper Casal, who described a disorder attributed to a diet deficient in meat [92]. Pellagra was epidemic in malnourished regions of Europe and the southern states of the United States of America. Pellagra has been characterised as a ‘disease with four D’s’—dermatitis, diarrhoea, dementia and death [93]. However, these classic symptoms rarely occur together or follow a predictable pattern, and are modified by environmental factors such as sun exposure, other concomitant vitamin deficiencies and disease progression [94]. Excessive alcohol consumption represents a major risk factor for pellagra and chronic alcohol use can induce niacin deficiency [95]. It has been reported that more than 100,000 Americans died from pellagra between 1907 and 1940. In 1914, Sir Joseph Goldberger linked pellagra to a nutrient deficiency due to a corn-rich diet. He suggested that dried yeast could be a cheap and effective therapeutic strategy to prevent pellagra [96]. However, it was not until 1937 that Dr. Conrad Elvehjem demonstrated that nicotinic acid (NA) and nicotinamide (NAM) cured pellagra [97]. 

The metabolic significance of nicotinamide adenine dinucleotide (NAD+) was first identified by Sir Arthur Harden in 1906, who demonstrated that boiling and filtering yeast extract enhanced alcoholic fermentation in unboiled yeast extract. The active component was termed extract conferment or cozymase [98]. In 1923, von Euler-Chelpin showed that cozymase was composed of a nucleoside sugar phosphate [99]. Subsequently, the role of NAD+ as a hydrogen carrier in anaerobic and aerobic oxidation was identified by Sir Otto Warburg in 1933 [100]. The pathways involved in NAD+ anabolism were fully characterised by Sir Arthur Kornberg, and the work of Priess and Handler in the 1940s and 1950s, respectively [101]. The redox roles of NAD+ in glycolysis, the tricarboxylic acid cycle, oxidative metabolism and energy production were elucidated by several scientists including Krebs. The non-redox roles of NAD+ as a substrate for ADP-ribosylation reactions and histone deacetylase activities have been elucidated in the last few decades [101]. Remarkably, maintaining NAD+ homeostasis is not only essential for the treatment of pellagra, but may also be associated with addiction, cardiovascular and neurodegenerative diseases and metabolic syndrome [54]. 

The significance of NAD+ in addictive disorders stems from the work of Dr. Paul O’ Hollaren (1961) who claimed to have successfully utilised IV NAD+ for the prevention and treatment of over 104 cases of addiction to alcohol and other drugs of abuse, including heroin, opium extract, morphine, dihydromorphine, meperidine, codeine, cocaine, amphetamines, barbiturates and tranquilisers [102]. In his retrospective case series, IV NAD+ was administered at a dose of 500–1000 mg added to 300 cc normal saline daily for 4 days, twice per week for a month, followed by a maintenance dose twice per month until addiction was ameliorated, with limited toxic effects [102]. NAD+ is likely to represent a cheap and useful holistic approach for the estimated millions of addicts worldwide, and may be an effective adjunct to psychotherapy, by ameliorating symptoms of physical addiction through a variety of mechanisms.

## 4. Summary of NAD+ Dependent Processes

There is a growing consensus that NAD+ levels decline at the cellular, tissue and organismal levels during ageing and progression of age-related degenerative diseases. NAD+ and its closely related phosphate NADP are cofactors in several anabolic and catabolic processes, such as fatty acid and cholesterol synthesis [103]. NAD+ and NADP, and their reduced forms, NADH and NADPH, are important cofactors in more than 400 enzymatic reactions including dehydrogenases, hydroxylases and reductases [104]. Recently, NAD+ has recently been reported to be an important reductant and hydride donor in biological oxidation of carbohydrate pathways [105]. NAD+ is essential for the dehydrogenation of acetylaldehyde, which is essential for alcohol metabolism. Impaired oxidative phosphorylation in rat brain cortical slices following exposure to acetylaldehyde was attenuated by the addition of NAD+ [106]. Although the reduced and phosphorylated forms can interconvert, they do not alter the levels of NAD+. The activity of several NAD-dependent enzymes or NAD+ ‘consumers’ are also affected by the decline in NAD+, influencing multiple processes and age-associated pathophysiologies. 

### 4.1. Poly(ADP-Ribose)Polymerases (PARPs)

Poly(ADP-ribose)polymerases (PARPs) are a family of DNA ‘nick’ sensors that detect DNA strand breaks through its N-terminal zinc-finger domain [107]. Poly(ADP)ribosylation breaks down NAD+ to NAM and an ADP-ribosyl product [108]. Oxidative damage and neuroinflammation have been reported parallel to DNA damage. Therefore, NAD+ depletion due to hyperactivation of PARP1 and 2 in the nucleus may play an important role in the pathology of central nervous system (CNS) disorders [109,110,111,112,113]. PARPs also regulate the tumour suppressor protein, p53. For instance, inhibition of poly(ADP)ribosylation and inactivation of p53 following treatment with etoposide was reported in PARP-deficient cell lines derived from Chinese hamster V79 cells [114]. PARP has also been reported to activate DNA-dependent protein kinases which influence p53 activity via phosphorylation [115]. Therefore, PARP activity is essential for the maintenance of DNA repair and genomic stability.

### 4.2. CD38/NAD+ Glycohydrolase

CD38 is a major NADase in mammalian cells, and another NAD+-consuming enzyme [116]. CD38 and CD157 hydrolyse NAD+ to generate ADP ribose (ADPR) and NAM. CD38 also produces the secondary messenger signalling molecule, cyclic-ADP-ribose (cADPR) which induces transient intracellular calcium waves. It has been estimated that about 100 molecules of NAD+ are necessary for hydrolysis to generate one molecular of cADPR. CD38 can also use the NAD+ precursors, nicotinamide mononucleotide (NMN) and nicotinamide riboside (NR) to regulate intracellular NAD+ levels [117]. It can also catalyse a base exchange between NADP and NA, generating nicotinic acid adenine dinucleotide phosphate (NAADP). CD38 has various immunomodulatory roles and increases in the levels of CD38 protein have been reported in several tissues over time, thus contributing to age-related NAD+ decline. CD38/cADPR also regulate oxytocin release, which is associated with social behaviours, and impaired CD38 function may induce several forms of neurological deficits [118]. 

### 4.3. Sirtuins

Silent information regulators of gene transcription, or sirtuins, are a family of class III NAD+ dependent histone deacetylases enzymes that translate changes in NAD+ levels to the regulation of several proteins associated with cellular metabolism, cellular stress responses, circadian rhythms and endocrine functions. The deacetylation reaction involves acetyl group transfer to ADPR to form a novel compound called 2′-*O*-acetyl-ADPR or acetylated ADP-ribose [119]. Cleavage of the glycosidic bonds in NAD+, leads to production of NAM as a by-product [120]. Mammalian cells have seven classes of sirtuins (SIRT1–7) located in various cellular organelles, and control a variety of important biological processes [121]. SIRT1 is a nuclear protein that is found mainly in the nucleus but can translocate to the cytoplasm, and is involved in promoting cellular longevity [122] by influencing the acetylation/deacetylation status of several important transcription factors, including the metabolic regulator, peroxisome proliferator-activated receptor-γ (PPARγ), tumour suppressor protein (p53) and the cell growth linked FOXO forkhead family of transcription factors [123]. SIRT2 is found in the cytoplasm but can also be found in the nucleus where it regulates gene expression and the cytoskeletal structure [124]. SIRT3 [125], SIRT4 [126] and SIRT5 [127] are found in mitochondrial compartments where they are involved in maintenance of mitochondrial redox status. SIRT6, another nuclear sirtuin, is involved in mediating an age-resistant phenotype [128]. Finally, SIRT7 is found in the nucleolus of mammalian cells where it mediates growth and metabolism [129]. Importantly, the beneficial effects of sirtuin activity are dependent on optimal NAD+ levels.

### 4.4. Sterile Alpha and Toll/Interleukin-1 Receptor Motif-Containing 1 (SARM1)

Sterile alpha and Toll/interleukin-1 receptor motif-containing 1 (SARM1) is a recently discovered NAD+ hydrolase. Axonal degeneration is associated with NAD+ depletion and inhibition of SARM1 function delays axonal degeneration. The Toll/interleukin-1 receptor (TIR) domain of SARM1 is dependent on NAD+ for NAD+ hydrolase activity and enhances axonal degeneration [130,131,132,133,134,135].

### 4.5. Interactions between NAD+ Consumers

We and others have previously demonstrated that the activity of PARP and SIRT1 is regulated by CD38, by potentially limiting the availability of NAD+ to its target enzymes [136]. NAM, which is also produced by the catalytic activity of CD38, is also an endogenous inhibitor of SIRT1 and CD38. Therefore, CD38 represents an important regulator of SIRT1 activity and SIRT1 functions, including maintenance of cellular bioenergetics, obesity and senescence, mainly because it modulates the availability of NAD+ to the SIRT1 enzyme [137]. There also exists a relationship between PARP1/2 and sirtuins, through their common substrate, NAD+. For example, PARP1/2 knockout enhances SIRT1 activity leading to increased mitochondrial function, fatty acid oxidation and protection against obesity [138,139]. However, while PARP1 knockout promotes NAD+ availability, PARP2 knockout promotes SIRT1 expression [140,141]. Therefore, chronic accumulation of oxidative stress and increased PARP activity plays a causal role in the decline in NAD+ and SIRT1 activity.

## 5. Overview of NAD+ Biosynthetic Pathways

**Figure 1:**
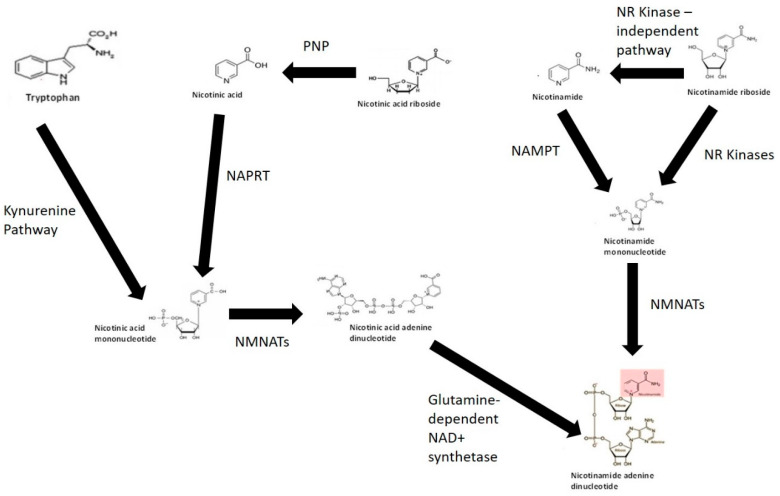
NAD+ biosynthesis pathways. The kynurenine pathway represents the de novo pathway of NAD+ is synthesis from catabolism of the amino acid tryptophan. NAD+ can also be synthesised via salvage of nicotinamide, nicotinic acid, nicotinic acid riboside and nicotinamide riboside form of vitamin B3. Abbreviations: NR kinase, nicotinamide riboside kinase; NAMPT, nicotinamide phosphoribosyltransferase; NMNAT, nicotinamide mononucleotide adenyltransferase; NAPRT, nicotinic acid phosphoribosyltransferase; PNP, purine nucleoside phosphorylase.

Given the importance of NAD+ in cellular bioenergetics and the need to maintain intracellular NAD+ pools in cells, several pathways are involved in the synthesis of NAD+. These include but may not be limited to: (1) de novo NAD+ synthesis from the amino acid TRYP via the KP; (2) NAD+ salvage pathway from vitamin B3 derivatives NA, NAM, NR and nicotinic acid riboside (NAR); and (3) major recycling pathway through NAM (Figure 1) (reviewed in [54]). Evidence suggests that the entire intracellular NAD+ pool may be consumed several times a day [142]. NAD+ anabolism is dependent on the availability of potential precursors, and not all of these precursors are bioequivalent [143].

### 5.1. NAD+ from Tryptophan

TRYP is converted to NAD+ via an eight-step process known as the KP. The conversion of TRYP to NAD+ occurs when the substrate supply is greater than the enzymatic capacity of 2-amino-3-carboxymuconate semialdehyde (ACMSD), an enzyme required for the synthesis of the NMDAR antagonist and endogenous metal chelator, picolinic acid (PIC) [54]. The NAD+ equivalence of 60 mg TRYP to 1 mg niacin may be partially explained by the fact that most of TRYP is used by the cell for protein synthesis. The daily recommend TRYP intake is 4 mg/kg body weight for adults [101]. Studies have shown that the levels of NAM have little effect on the de novo biosynthesis rate of NAD+ from TRYP. Oral supplementation with TRYP at 15 g/day has been reported to induce some adverse effects including drowsiness and headache [144]. Overconsumption of TRYP is capable of increasing the levels of QUIN which has been associated with neurodegenerative disorders, anxiety and seizures [48]. Therefore, TRYP is unlikely to represent an ideal pharmaceutical source to raise NAD+ levels.

### 5.2. NAD+ from Nicotinic Acid

NA is converted to nicotinic acid mononucleotide (NAMN) by the ATP-dependent enzyme nicotinic acid phosphoribosyl transferase (NAPRT) (EC 6.3.4.21) using PRPP as a co-substrate. As QUIN is converted to NAMN by the enzyme QPRT, the sequence of events leading to NAD+ production is identical to NAD+ from TRYP after NAMN formation [145]. NAPRT appears to be expressed in several catabolic tissues including the colon, heart, kidney and liver [146]. Adenylation of NAMN is dependent on the catalytic activity of NAD pyrophosphorylase or nicotinamide mononucleotide adenylyltransferase (NMNAT) (EC 2.7.7.1) in the presence of ATP to produce desamido NAD [147,148]. Three NMNAT isoforms are NMNAT-1 (nucleus), NMNAT-2 (Golgi complex) and NMNAT-3 (mitochondria) [149]. This suggests an organelle-specific function for enzymes, and specific nuclear, mitochondrial and Golgi-specific NAD+ anabolic pathways. NMNAT-2 and -3 can also form NADH directly from reduced nicotinamide mononucleotide (NMN) [150]. NMNAT activity (and predominantly NMNAT-1) is high and non-rate-limiting in catabolic tissue, but not in blood [151].

The process involved in the synthesis of NAD+ from NA is known as the Priess–Handler process. NA has been shown to increase intracellular NAD+ levels in a kidney cell line [152]. Oral supplementation of 1–3 g of NA daily lowers blood triglyceride levels and low-density lipoproteins (LDLs), and increases the levels of high-density lipoproteins (HDLs) [153,154]. Exogenous NA also increases intracellular NAD+ levels in brain cells [155]. However, NA therapy induces significant skin flushing in most individuals, thus limiting its clinical use as an NAD+ precursor. A mild skin flush has been reported in patients exposed to doses as low as 50 mg oral NA [156]. The lipid lowering effects and adverse effects of NA are thought to be mediated by interaction of NA to the cell surface of a G-protein coupled receptor known as HM74A or GPR109A, which enhances the conversion of the omega-6 metabolite arachidonic acid (AA) into prostaglandin E2, stimulating vasodilation of skin capillaries, causing unwanted skin flush [156].

### 5.3. NAD+ from Nicotinamide Recycling

Data in the literature suggest that half-lives of most enzymes involved in NAD+ consumption are 4–10 h [142]. This suggests that 200–600 µmol/kg of NAM needs to be recycled back to NAD+ per day per tissue in rats. This is equivalent to consumption of 3 g of NAM several times a day in a 75 kg adult human [142]. These levels are considerably lower than the recommended doses of NAM from diet (i.e., 1 lb tuna is required for 100 mg NAM, 1 lb beef generates 30 mg NAM while four cups of broccoli retain 4 mg NAM). Therefore, effective NAM recycling pathways are necessary to protect against NAD+ deficiency in humans. Recycling of NAM to NAD+ is dependent on the enzyme nicotinamide phosphoribosyl transferase (NAMPT) (EC 2.4.2.12) using PRPP as a co-substrate which converts NAM to NMN, and then to NAD+ by the action of NAD pyrophosphorylases in the presence of ATP [157]. The rate of NAM recycling is highest in the liver and kidney, and lowest in blood [151]. The recommended dose of NAM is 14–16 mg per day and is suggestive of a net loss not exceeding 0.5% total NAD+ per day to maintain NAD+ homeostasis. Considering that the intracellular NAD+ pools may be replaced up to four times per day, this reflects a total loss of 0.1–0.2% NAM per cycle [158]. NAM is methylated by the enzyme nicotinamide N-methyltransferase (NNMT) (EC 2.1.1.1) to N-methylnicotinamide (MeNAM). MeNAM plays important roles in several metabolic and epigenetic processes and can influence neurodegeneration and ageing. Therefore, while supplementation with NAM can raise NAD+ and does not cause flushing, it is not considered an ideal supplement due to its enzyme inhibiting (e.g., PARPs, sirtuins, CD38) and methyl depleting potential.

### 5.4. NAD+ from Nicotinamide Riboside and Nicotinic Acid Riboside

NR or NAR represent newly identified precursors that can be used to synthesise NAD+ via the NR kinase (NRK) (EC 2.7.1.173) pathway [159]. Two NRK enzymes have been identified, NRK1 and NRK2, although, their exact physiological roles remain unclear. Dephosphorylation of NMN into NR, which is required to produce NAD+ in yeast, represents a crucial step for increasing intracellular NAD+ in mammalian cells [160]. NR can also be catabolised into a ribosyl product and NAM via an NRK-independent pathway, which can then be further recycled to yield NAD+. Purine nucleoside phosphorylase (PNP) (EC 2.4.2.1) can convert NAR to NA, which is then converted to NAMN by the activity of NAPRT [161]. NR appears to be safe and orally bioavailable in mice and humans with very few minor side effects reported [162]. NR is currently a lead candidate in several preclinical and human clinical trials to evaluate whether it can be used for the treatment of age-related degenerative disorders [163], given that NAD+ decline and/or increased NAD+ consumption may be a major risk factor in these debilitating disorders.

## 6. NAD+ Metabolism: Cellular Energy, Secondary Messenger Signalling and Manipulation for Addiction

Given the global effects of NAD+ and SIRT1 on human physiology and function, NAD+ levels can influence anxiety, exploratory and depressive behaviour, and the brain reward system linked to addiction. NAD+ is likely to represent a molecular link between metabolism and psychiatric conditions [164]. It has been well established that substance abuse and food addiction can impair metabolism, alter the cellular redox status, disrupt circadian rhythms and promote oxidative stress and neuroinflammation [165]. NAD+ regulates intracellular calcium levels during DA release via increased CD38 activity, therefore reducing NAD+ availability [166]. There is also evidence of a functional relationship between NAD+ and DA levels in the brain which is likely to be critical to brain function in physiological and pathological settings [101,167,168]. Mechanisms by which NAD+ and its related processes can influence the neurobiology of addiction are described below.

### 6.1. SIRT1 Regulates Behavioural Responses Associated with Drug Addiction 

The crucial role for SIRT1 and SIRT2 in modulating behavioural responses to cocaine and morphine in the NAc has been previously studied. One study reported increases in SIRT1 and SIRT2 expression in the mouse NAc following chronic cocaine administration, while only SIRT1 expression was increased following chronic morphine administration [169]. Interestingly, cocaine and morphine had no effect on the expression of other sirtuin family members. Increased expression of SIRT1 and SIRT2 following exposure to drugs of abuse is partly attributed to the drug-induced transcription factor ΔFosB. In addition, the rewarding effects of cocaine and morphine were enhanced in mice following viral-mediated overexpression of SIRT1 or SIRT2 in the NAc and localised knockdown of SIRT1 in the NAc reduced drug reward [169]. Therefore, sirtuins and SIRT1 and SIRT2, play key roles in mediating alterations in molecular and cellular plasticity induced by drugs of abuse in NAc, and may regulate behavioural adaptations following exposure to substance abuse.

The exact mechanisms by which SIRT1 can mediate cocaine-induced plasticity in NAc remain unclear. One study used chromatin immunoprecipitation after repeated cocaine (20 mg/kg) or saline injections, to characterise the SIRT1 binding genome-wide in mice NAc [170]. The study reported three major findings. Firstly, chronic cocaine causes depletion of SIRT1 from most affected gene promoters and increases in H4K16ac (a deacetylation substrate of SIRT1). Secondly, cocaine-induced SIRT1 induction in the NAc promotes deacetylation and activation of FOXO3a and upregulation of various FOXO3a gene targets in other systems. Thirdly, overexpression of FOXO3a in NAc promotes cocaine place conditioning [170]. Taken together, these findings suggest that SIRT1, an NAD-dependent enzyme, can influence molecular adaptations associated with cocaine addiction.

### 6.2. NAD+ and SIRT1 Regulate Diurnal Rhythms Associated with Addiction Behaviour

**Figure 2:**
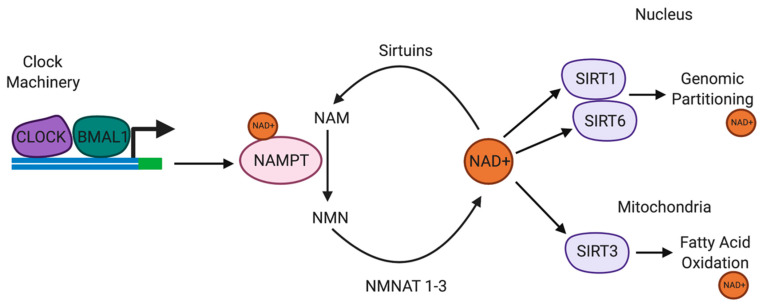
Circadian rhythms are regulated by the availability of NAD+. NAMPT is a SIRT1/CLOCK/BMAL1-regulated circadian gene. SIRT1 and NAMPT and NMNAT form part of a circadian regulatory feedback loop, regulating the availability of NAD+. NAD+ regulates SIRT1, SIRT3 and SIRT6 activities. SIRT1 also regulates CLOCK/BMAL1 expression in the suprachiasmatic nucleus. SIRT6 regulates chromatin recruitment of CLOCK/BMAL1. SIRT3 regulates oxidative phosphorylation and fatty acid oxidation in the mitochondria via circadian deacetylation of mitochondrial enzymes. Abbreviations: NAD+, nicotinamide adenine dinucleotide; NAMPT, nicotinamide phosphoribosyltransferase; NMNAT, nicotinamide mononucleotide adenyltransferase; SIRT, sirtuins.

The circadian transcription factors CLOCK and BMAL1 form heterodimers bind to enhancer E-box promoter elements to directly regulate gene transcription (Figure 2) [171]. Owing to the location of E-box promoter elements to the transcriptional start site and the cAMP response element (CRE), direct transcriptional regulation of TH is mediated by CLOCK/BMAL1 [172]. TH is the enzyme responsible for catalysing the synthesis of L-3,4-dihydroxyphenylalanine (L-DOPA) from the amino acid L-tyrosine, using molecular oxygen (O_2_), iron (Fe^2+^) and tetrahydrobiopterin as cofactors [173]. L-DOPA is a precursor for DA, which is essential for the synthesis of the vital neurotransmitters norepinephrine (noradrenaline) and epinephrine (adrenaline) [173]. TH enzyme represents the rate limiting step in the synthesis of catecholamines and is encoded by the TH gene. The TH gene is expressed in the CNS, peripheral sympathetic neurons and the adrenal medulla [173]. 

CLOCK is disrupted in substance abuse and mice exhibiting mutations in CLOCK are hyperdopaminergic and more susceptible to drug addiction [174]. TH expression is upregulated in the mesolimbic DA system in mice harbouring the CLOCKΔ19 mutation, providing evidence of the regulatory role of CLOCK on the transcriptional activity of TH [175]. Recent evidence suggests that the interaction between CLOCK and BMAL1 is regulated by the NAD-dependent histone deacetylase enzyme SIRT1 to repress CLOCK-mediated transcription [176]. A recent study showed that CLOCK and NAD-dependent SIRT1 antagonise CREB-mediated transcription during the day, which is attenuated during the night, thus facilitating CREB-induced TH transcription at night. Impaired CLOCK in CLOCKΔ19 mice induces CREB-induced TH transcription during the day [176]. 

Nicotinamide phosphoribosyltransferase (NAMPT) is a rate-limiting enzyme in NAD+ biosynthesis pathway. NAMPT catalyses the conversion of NAM into NMN, which is subsequently converted to NAD+. One study recently demonstrated that NAMPT expression was significantly upregulated in the ventral tegmental area (VTA) of cocaine-conditioned mice [177]. Intraperitoneal or intra-VTA injection of the NAMPT inhibitor FK866, attenuated cocaine reward, although the effect was repressed by increasing intra-VTA expression of NAMPT or supplementation with NMN [177]. The levels of NAD+ and NMN, and SIRT1 expression were elevated in the VTA in cocaine-conditioned mice. However, the inhibitory effect of FK866 on cocaine reward was markedly reduced in SIRT1 midbrain conditional knockout mice [177]. These results suggest that NAMPT-mediated NAD+ biosynthesis may influence cocaine behavioural effects via SIRT1. 

NMN, an NAD+ precursor, also reduced CREB-mediated transcription, providing support for the importance of maintaining NAD+ levels as a key regulator of SIRT1-mediated transcriptional repression. Interestingly, the recycling of NAM to NMN is regulated by the CLOCK target gene, NAMPT [176]. NAMPT and SIRT1 expression are highest during the diurnal phases and are opposite to the levels of NAD+ and SIRT1 protein, suggestive of NAD-dependent SIRT1 mediated repression of TH expression during the inactive phase. Daily changes in NAD+ levels regulate the CLOCK/BMAL1/SIRT1 heterodimer complexes [178]. More specifically, CLOCK/SIRT1 preferentially binds to the TH promoter during the day when the levels of NAD+ are highest and the expression of TH is lowest. 

Upregulation of SIRT1 and SIRT2 activities appears to be associated with increased excitability of NAc neurons following repeated cocaine exposure [179]. This raises the possibility for the application of SIRT1 and SIRT2 inhibitors as potential agents to treat cocaine addiction.

### 6.3. NAD+ Increases Adenosine Levels Which Counteract the Effects of Dopamine

The modulatory effects of DA are mediated by postsynaptic D1 and D2 receptors. The neuromodulator, adenosine, has been reported to attenuate DA signalling via modulation of adenosine A1 and A2A receptors. Thus adenosine receptors serve as endogenous ‘brakes’ on overactivated DA receptors and can attenuate drug-induced changes to neuronal function and cognitive disorders [180]. Adenosine A1 receptors are found in the presynapsis where they compete with A2A receptors by receptor–receptor mediated interactions to inhibit glutamate release. Adenosine A1 and DA D1 receptors are expressed in the post synapsis on dynorphin-containing neurons while adenosine A2A receptors are expressed with dopamine D2 receptors in encephalin containing neurons [181]. These receptors mediate opposing effects including allosteric receptor-receptor interactions and intracellular signalling cascades. Drugs of abuse have been reported to alter the expression of adenosine and DA receptors in the mesolimbic DA system, this limiting adenosine receptor activity in favour of increased DA stimulation and promoting addictive behaviour although the exact mechanism(s) remain unclear [182].

Treatment with exogenous NAD+ has been shown to increase adenosine levels which are likely to activate adenosine receptors to counteract the degenerative effects of DA on neuronal cells [183]. Endogenous adenosine levels have been reported to be in the nanomolar range [184], and the potential beneficial effects of NAD+ in addiction may be partially mediated by increased adenosine levels and activation of adenosine receptors. A recent study showed that treatment with exogenous NAD+ increased intracellular adenosine levels in BV2 microglial cells which could enter cells through equilibrative nucleoside transporters [185]. Increases in the intracellular adenylate pool can be converted to AMP by the catalytic activity of adenosine kinase, which increases intracellular ATP levels by enhancing AMP kinase (AMPK) activity and increasing ADP to promote mitochondrial ATP synthase activity [186]. Therefore, extracellular NAD+ may have beneficial effects in addiction therapy through its degradation into adenosine, attenuation of DA receptor activity and the activities of adenosine kinase and AMPK.

### 6.4. NAD+ and SIRT1 Regulate Monoamine Oxidase A

It has been reported that SIRT1 regulates anxiety and addictive behaviour, although the exact mechanism remains unclear [187,188,189,190,191]. It has been recently reported that the NAD-dependent SIRT1 deacetylates the brain-specific transcription factor NHLH2 on lysine 49, which leads to increases in the activity of the monoamine oxidase A (MAO-A) promoter, thus activating MAO-transcription [190]. Since MAO-A is responsible for the degradation of serotonin, increased MAO-A leads to reduced serotonin levels, thus increasing anxiety and depression. MAO-A inhibitors have been reported to normalise anxiety differences in murine models exhibiting altered brain SIRT1 levels. Genetic analysis of unbiased human cohorts reported that the role of sirtuins in regulating anxiety and behavioural disorders is conserved. These studies provide evidence for the role of sirtuins and the essential substrate NAD+ in mediating psychological effects on stress-response pathways [190].

### 6.5. NAD+ Regulates FGF21 and Oxytocin Signalling via SIRT1 and CD38

NAD-dependent SIRT1 plays an important role in regulating cellular energy homeostasis. In peripheral tissue, SIRT1 promotes the use of fat as a substrate [192,193,194,195,196]. On the other hand, brain SIRT1 facilitates homeostatic feeding control enhancing hormone sensing. Recently, SIRT1 has been reported to be a regulator of macronutrient preferences and regulates macronutrient preference via FGF21-Nrf2-OXT signalling [197]. FGF21 enhances OXT-mediated neuronal activation via ERK signalling and regulates OXT expression via AKT signalling. SIRT1 promotes FGF21 sensitivity in OXT neurons and enhances negative feedback processes that regulate simple sugar preference [197]. 

Importantly, obesity is associated with FGF21 resistance in peripheral tissue and the CNS due to reduced FGF21 receptor expression. FGF21 resistance in the CNS may play a critical role in obesity and metabolic syndrome [198]. Hepatic SIRT1 is a key regulator of FGF21 transcription. Increased systemic SIRT1 activity due to increased NAD+ levels can enhance FGF21 signalling by raising the levels of FGF21 from the liver and increasing FGF21 signalling in OXT neurons [199]. Interestingly, the circulating levels of OXT are reduced in obesity, and intranasal treatment with OXT led to a metabolic shift from using carbohydrates to fat [197,200]. Given the brain FGF21 stimulates OXT neurons and inhibits simple sugar preference, increasing FGF21 levels may also improve food selection and improve health and promote stress resistance. 

**Figure 3:**
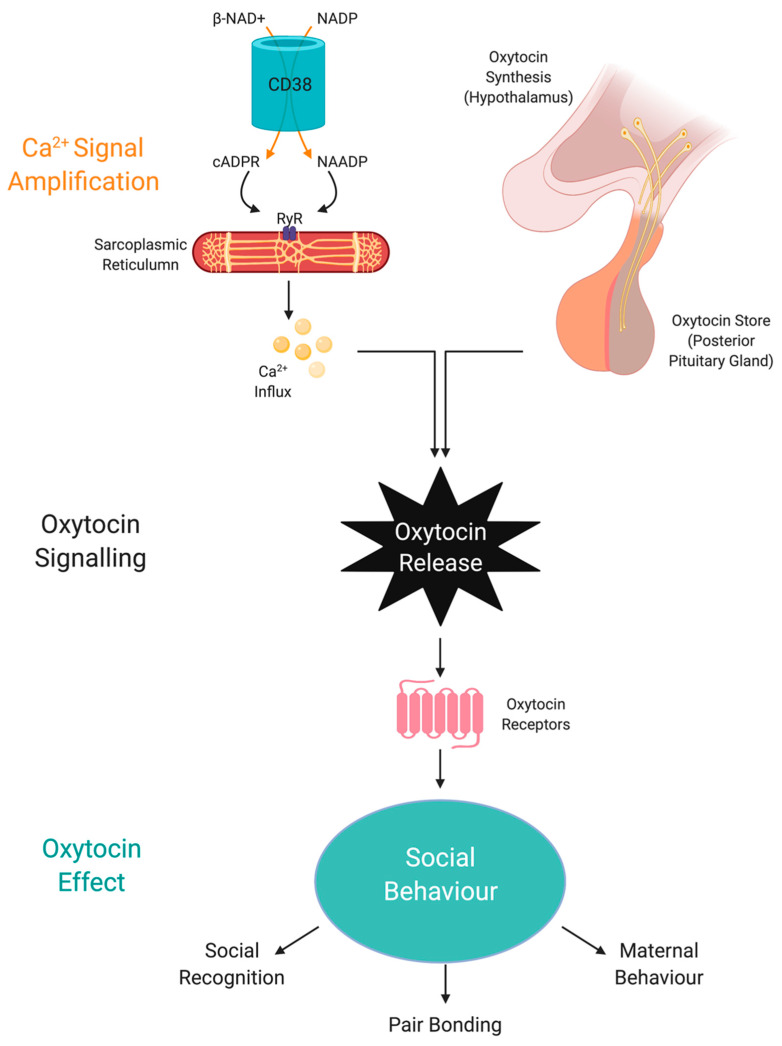
NAD+ regulates oxytocin activity via CD38 and cADPR. Social behaviour is regulated by the amount of central OXT released due to NAD-dependent calcium release by CD38 activity and cADPR generation. Abbreviations: NAD+, nicotinamide adenine dinucleotide; NADP, nicotinamide adenine dinucleotide phosphate; CD38, NAD+ glycohydrolase; cADPR, cyclic adenosine diphosphoribose; OXT, oxytocin; NAADP, nicotinic acid adenine dinucleotide phosphate; Ryr, ryanodine receptor.

Apart from SIRT1, NAD+ can also regulate OXT through CD38 [201]. Effective social behaviour is dependent of optimal CD38 activity to regulate OXT secretion from the hypothalamus and pituitary [202] (Figure 3). Social amnesia may be induced by reducing OXT secretion from the hypothalamus when CD38 activity is reduced due to gene manipulation or reduced availability of NAD+ [203]. 

### 6.6. NAD+ and SIRT1 Regulate Drp1 and Mitochondrial Fission to Potentiate Drug Seeking

**Figure 4:**
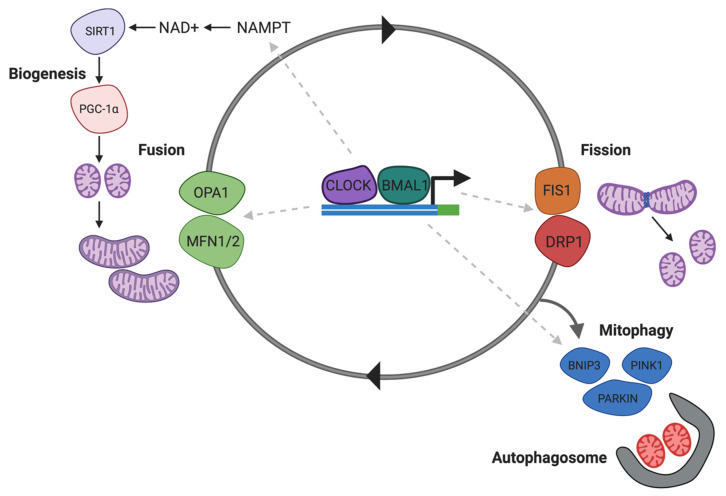
NAD+ regulates Drp1 associated in mitochondrial homeostasis. CLOCK/BMAL1 modulated mitochondrial biogenesis and mitophagy via NAD-dependent SIRT1. Deacetylation of the transcription factor PGC1A by SIRT1 regulates mitochondrial biogenesis. Drp1-mediated mitochondrial fission precedes mitophagy leading to formation of fragmented mitochondria that are taken up by autophagosomes. Abbreviations: NAD+, nicotinamide adenine dinucleotide; NAMPT, nicotinamide phosphoribosyltransferase; SIRT1, sirtuin-1; OPA1, mitochondrial dynamin like GTPase; MFN1/2, mitofusin-1/2; FIS1, mitochondrial fission 1 protein; DRP1, dynamin-related protein-1; PINK1, PTEN-induced kinase 1; BNIP1, BCL-2 interacting protein 1; PARKIN, E3 ubiquitin-protein ligase parkin.

Impaired energy metabolism has been reported in the brains of subjects addicted to substances, and cocaine, although the exact mechanism remains unclear. It has been recently demonstrated that the dynamin-related protein-1 (Drp1), which mediated mitochondrial fission, is increased in the NAc following chronic cocaine exposure [204]. A Drp1 inhibitor, Mdivi-1 inhibited cocaine seeking behaviour, and antagonised c-Fos induction and excitation onto D1 DA receptors [204]. Since Drp1 expression and mitochondrial fission are regulated by SIRT1 [205] and NAD+ (Figure 4) by deacetylating p53, reduced SIRT1 activity and NAD+ availability can alter brain energy homeostasis affecting behavioural and cellular plasticity during addiction. 

## 7. Is There Room for NAD+ in Addiction Therapy?

While NAD+ precursors (e.g., NAM, NMN and NR) have been shown to increase NAD+ levels after treatment, IV NAD+ remains the most direct method of raising NAD+ levels, although it is yet to gain U.S. Food and Drug Administration (FDA) approval. While data from experimental studies are limited, significant clinical benefit of IV NAD+ infusion in alcohol and opioid withdrawal has been previously reported [102]. These authors found significant improvements in the removal of cravings and withdrawal symptoms. The benefits of NAD+ are likely to be extended to improving emotional disorders in patients with psychological disorders. Whilst there has been growing enthusiasm for the benefits of IV NAD+ therapy, the pharmacokinetics and pharmacodynamics of IV NAD+ remain nascent in the current literature. 

A recent study documented changes in levels of NAD+ and key metabolites in the NAD+ metabolome (henceforth the NADome) in both plasma and urine over 8 h using a typical clinical dosing regimen of 750 mg NAD+ administered IV over a 6 h period [206]. The study was the first to report that: (a) the level of NAD+ remained constant in the plasma for at least the first 2 h when administered at a flow rate of 3 μmole/min; (b) increases in the levels of metabolic bi-products analysed were consistent with the activity of NAD+ consumers (e.g., PARPs, CD38 and sirtuins); and (c) by-products of NAD+ metabolism and unbound NAD+ itself can be excreted in urine [206]. However, further research is necessary to fully understand the complex metabolic fate of NAD+ following treatment.

Importantly, no adverse effect was reported following IV NAD+ infusion when administered at an appropriate rate [102,206]. However, reduced plasma activities of enzymes associated with hepatic stress such as the intrahepatic LD and AST and the post-hepatic (bile duct) enzyme GGT suggest that NAD+ is essential for the maintenance of structural and functional integrity of both intra-hepatic and post-hepatic tissue. Increases in bilirubin, a red cell degradation product, after 8 h may be indicative of a minor increase in red cell turnover, possibly due to infusion-induced haemolysis, or reduced heme metabolism. Since these increases are in a low magnitude of change, it was not considered to be of clinical relevance [206]. IV NAD+ therapy may provide significant improvement in addictive disorders likely due to increased NAD+ availability. Further longitudinal and follow-up studies are necessary to cement the role of IV NAD+ in addiction therapy. In addition, the effect of oral administration of the NAD+ precursors, NMN and NR, as potential therapeutic agents for raising NAD+ and improving addictive symptoms warrants further clinical investigation. 

## 8. Conclusions 

The central role of NAD+ in the maintenance and regulation of cellular bioenergetics, and modulation of numerous secondary messenger signalling pathways suggest that NAD+ is essential for promoting cellular regeneration and repair in neuropsychiatric conditions such as addiction, Alzheimer’s disease and other neurodegenerative dementias. A growing body of evidence suggests that NAD+ levels decline with age and an imbalance in NAD+ anabolism and NAD+ consumption can lead to a significant derailment of fundamental molecular processes leading to accelerated degeneration and ageing [163,164,207]. This review attempts to provide renewed insight into current knowledge of the NADome, and mechanisms by which NAD+ can interact with various processes in cells and tissues to attenuate addictive behaviour and reduce the addictive phenotype in the clinic. If NAD+ anabolism is impaired and/or if NAD+ consumption is increased during addiction, this is likely to reduce intracellular NAD+ pools which contribute to functional decline. NAD+ consumers such as PARPs, CD38, sirtuins and the more recently identified SARM1, may be affected not only in the brain but in other cells and tissues leading to a loss in NAD+ homeostasis that plays a contributory, if not causal, role in deficits in basic physiological processes associated with the neurobiology of addiction. 

For several decades, IV NAD+ has been used as a holistic ‘underground’ approach for the treatment of various forms of addiction. IV NAD+ showed complete withdrawal of addictive drugs without subjects experiencing ‘agony of withdrawal’ symptoms [102,206]. It also provides a cheap and direct means for physicians to treat addiction without the necessity of using synthetic therapeutic agents that are more costly, pose greater risk of adverse effects and induce addiction or regimens that require ‘trafficking’ of addictive drugs for gradual withdrawal. However, it should be clarified that the use of NAD+ does not provide allowance for continued use of addicted drugs. Rather, NAD+ therapy is aimed at improving ‘productive ageing’ by restoring health processes, removing cravings and withdrawal symptoms and assisting in abstinence. Emerging studies using IV NAD+ in addiction set the stage for deeper investigations into the mechanisms by which addiction is sensitive to NAD+ status, and how it can improve, if not attenuate, addiction in the clinic. 

Apart from the historical benefits of niacin and IV NAD+ in addiction therapy, the identification of NR, NAR and NMN as NAD+ precursors provide ‘newer’ alternatives for raising NAD+ levels and to possibly alleviate biochemical processes associated with addiction. These NAD+ precursors have already demonstrated protective effects in several animal models of disease including neurodegenerative disorders, cardiovascular disease, metabolic syndrome, cancer and ageing [54,163]. Given recent advances in quantifying the NADome in various biological specimens using specific biosensors and mass spectrometry techniques [208], we are at an exciting time when we can evaluate the importance of NAD+ metabolism not only for prevention and management of ageing and age-related disorders, but in addictive behaviour as well.
